# Warm homes for older people: aims and methods of a randomised community-based trial for people with COPD

**DOI:** 10.1186/1471-2458-13-176

**Published:** 2013-02-26

**Authors:** Helen Viggers, Philippa Howden-Chapman, Tristram Ingham, Ralph Chapman, Gina Pene, Cheryl Davies, Ann Currie, Nevil Pierse, Helen Wilson, Jane Zhang, Michael Baker, Julian Crane

**Affiliations:** 1 He Kainga Oranga/Housing and Health Research Programme, University of Otago, PO Box 7343, Wellington, Wellington South, New Zealand; 2 Department of Medicine, University of Otago, PO Box 7343, Wellington, Wellington South, New Zealand; 3 School of Geography, Environment and Earth Sciences, Victoria University of Wellington, Wellington, New Zealand; 4 Tu Kotahi Māori Asthma Trust, 7-9 Barnes St, Seaview, Lower Hutt, New Zealand; 5 Community and Public Health, Canterbury District Health Board, 310 Manchester St, Christchurch, New Zealand

**Keywords:** New Zealand, COPD, Housing, Health, Fuel poverty, Randomised community trial

## Abstract

**Background:**

Chronic Obstructive Pulmonary Disease (COPD) is of increasing importance with about one in four people estimated to be diagnosed with COPD during their lifetime. None of the existing medications for COPD has been shown to have much effect on the long-term decline in lung function and there have been few recent pharmacotherapeutic advances. Identifying preventive interventions that can reduce the frequency and severity of exacerbations could have important public health benefits. The Warm Homes for Elder New Zealanders study is a community-based trial, designed to test whether a NZ$500 electricity voucher paid into the electricity account of older people with COPD, with the expressed aim of enabling them to keep their homes warm, results in reduced exacerbations and hospitalisation rates. It will also examine whether these subsidies are cost-beneficial.

**Methods:**

Participants had a clinician diagnosis of COPD and had either been hospitalised or taken steroids or antibiotics for COPD in the previous three years; their median age was 71 years. Participants were recruited from three communities between 2009 to early 2011. Where possible, participants’ houses were retrofitted with insulation. After baseline data were received, participants were randomised to either ‘early’ or ‘late’ intervention groups. The intervention was a voucher of $500 directly credited to the participants’ electricity company account. Early group participants received the voucher the first winter they were enrolled in the study, late participants during the second winter. Objective measures included spirometry and indoor temperatures and subjective measures included questions about participant health and wellbeing, heating, medication and visits to health professionals. Objective health care usage data included hospitalisation and primary care visits. Assessments of electricity use were obtained through electricity companies using unique customer numbers.

**Discussion:**

This community trial has successfully enrolled 522 older people with COPD. Baseline data showed that, despite having a chronic respiratory illness, participants are frequently cold in their houses and economise on heating.

**Trial Registration:**

The clinical trial registration is http://NCT01627418

## Background

Reducing the impacts of chronic diseases is a growing problem. By 2030, Chronic Obstructive Pulmonary Disease (COPD) is projected to become the third most common cause of death globally with about one in four people estimated to be diagnosed with COPD during their lifetime [1,2]. The main characteristics include a progressive, irreversible decline in lung function, accompanied by breathlessness, a chronic cough and increased sputum production. Exacerbations, or acute worsening of the symptoms, which lead to a decrease in health-related quality of life, are part of the natural history of the disease [3]. While smoking is a major cause, there is a strong social gradient in COPD mortality, independent of smoking [4,5].

None of the existing medications for COPD has been shown to have much effect on the long-term decline in lung function and there have been few pharmacotherapeutic advances [6]. Identifying preventive interventions that can reduce the frequency and severity of exacerbations could have important public health benefits.

### The cost of COPD to society

COPD is a leading reason for admission to hospital, acute visits to outpatient and emergency departments and family doctors. It is estimated that about half the average COPD costs are due to hospitalisation and the annual hospitalisation costs for a severe COPD patient are about six times greater than those for a patient with mild or moderate COPD [7]. National direct and indirect annual costs vary from approximately $US37.2 billion in an American study [8] to between $A0.8 and $A1.0 billion per year in an Australian study [9].

In New Zealand, COPD is among the top four major causes of avoidable hospitalisation, with COPD-related hospitalisations rising rapidly over the last two decades. Depending on assumptions about costings and average length of stay, direct treatment cost estimates range from $NZ16.4 million in 1998/9 [10], to $NZ102-192 million in 2003 [1].

### COPD and housing

A number of studies have linked socio-economic status (SES) with respiratory illness [11] and specifically COPD [4,12-15], but few studies have looked at possible links between housing conditions and exacerbations of COPD. A review of socio-economic determinants of COPD [16] looked at home conditions and behaviours, but only one paper specifically included a housing quality measure (summarised as years without central heating) which was significantly inversely related to lung function [17].

Studies relating housing and health often use housing as a marker for SES, rather than exploring the role of the housing itself [18]. In addition, studies that have examined the role of housing in COPD often use only a single housing variable. Three studies, using different housing measures - a composite variable (number of rooms and sector of landlord) [19]; two binary indicators of housing quality (presence of central heating and one or more rooms per person) [4]; and the number of people living at home [12] -- did not find any observed relationship with COPD. One set of papers [20,21] followed COPD patients through an energy efficiency upgrade; this is reviewed further below.

A review looking at improving life for COPD patients only mentioned the home in terms of therapies taken there [22]. Another review found that only one of 171 papers referred to the home environment (temperature) [23]. Nonetheless, Burge suggests the way forward is a combination of interventions including warmer housing [24].

### Temperature and respiratory illness

There is a clear association between a cold environment and morbidity both generally [25] and for people with COPD [26] during winter in particular [27,28]. COPD patients who experience frequent exacerbations are more likely than those with less frequent exacerbations to acquire a cold, but both groups have approximately the same rates of COPD exacerbations once the cold has been acquired [29,30]. Therefore, reducing the risk factors associated with common winter respiratory diseases might have a disproportionately advantageous effect on individuals with COPD.

In New Zealand, the excess winter hospitalisation rate for COPD is 70% (rate ratio 1.70, 95% CI 1.67, 1.73 for 2000–2004) (Zhang, Viggers et al., submitted). New Zealand also has a greater seasonality of mortality, driven by effects on those over 65, than the more extreme climates of the United Kingdom, the United States, Australia, Japan or Sweden [27,31]. People over the age of 80 have an approximately 74% increased chance of dying of respiratory disease in winter rather than other seasons [31,32]. A study linking census and mortality data showed an increased risk of dying in winter among low-income people, those living in rented accommodation and those living in cities [33]. Such excess winter mortality also applies to cardiovascular diseases, but not to non-housing related diseases, such as cancer. These findings suggest modifying people’s heating behaviour in winter might result in reduced rates of illness and possibly mortality.

### Temperature of New Zealand homes

Compared to other countries with similar climates, houses in New Zealand are colder, with an *average* winter evening living room temperature of 17.9°C and a mean range of 10°C- 23.8°C [34]. Often only one room, usually the living room, is heated during winter [35]. Average winter overnight bedroom temperatures fall below 14°C [36], indicating that many bedrooms are colder than this for all or part of winter.

The World Health Organization included the elderly in groups vulnerable to low indoor temperatures in the home, and stated that below 16°C there was a serious risk to health, and between 16°C and 19°C there were small risks to health after substantial periods of time [37]. In England and Wales if people are home all day acceptable temperatures are defined as 21°C in the main living area and 18°C in other occupied rooms [38], but the New Zealand Building Code’s only relevant specification is that the interior temperature of old people’s homes and early childhood centres should be able to be maintained at a minimum of 16°C [39].

There are suggestions that higher morbidity, as well as mortality, may be compounded by energy-inefficient or poorly heated housing, particularly for older people [40-43]. Research we have previously conducted found that insulating houses led to a significantly warmer, drier indoor environment and resulted in improved health and a trend for fewer hospital admissions for respiratory conditions [44,45]. The addition of more effective, sustainable heating, as well as retrofitted insulation, in houses where there were children with asthma problems, reduced asthma symptoms, visits to the doctor and school absences [46].

### Fuel poverty and increasing electricity prices

Poorly heated housing is influenced by three factors: the thermal quality of the house; the income of the occupants; and the price of fuel [47]. Britain has developed comprehensive policies to address fuel poverty, defined as households where more than 10% of income must be spent to achieve adequate temperatures, although that definition is currently subject to debate [48,49]. Policies include a universal non-means-tested, non-taxable Winter Fuel Allowance for households containing people aged over 60, improvement in the quality of social housing, and energy efficiency measures [38,50].

A model of the effect of the Winter Fuel Allowance [51] drew on the concept of mental accounting, a term coined by Thaler to describe models and rules that individuals use to organise their finances [52,53]. Munro and colleagues hypothesised that while there was no requirement that the Fuel Allowance *would* be spent on fuel bills, there was some evidence that money labelled as a ‘fuel allowance’ and nominally allocated to this particular area of expenditure was more likely to be spent there, so may be an effective way to increase warmth. Moreover, English households that receive heating and insulation interventions have shown greater increases in warmth than those that received insulation interventions alone [54].

Fuel poverty has been established as an increasing problem in New Zealand, with potentially 25% of households meeting this definition in 2008, up from 12% in 2001. With the rising price of energy, its incidence and severity are increasing [55]. In the last decade, nominal average retail electricity prices increased over 80% [56], and real prices by 40%.

The cost of electricity has been rising faster than inflation-adjusted superannuation leading to particular difficulties for older people, who may be on fixed incomes and lack the physical strength to use fuels such as wood or wood pellet bags [57]. The use of wood heaters is also problematic for COPD patients as indoor air pollution can trigger exacerbations [58].

The Ministry of Social Development’s Economic Living Standard Index, which provides a scale applicable to the general population, has two items pertinent to fuel poverty: “put up with cold” and “stayed in bed for warmth” [59]. This underlines that among poorer New Zealanders domestic warmth is not always affordable.

### Housing interventions for people with COPD

Possible links have long been made between cold housing and COPD exacerbations for example, in indoor environments which are cold with high relative humidity, mattresses can become very damp and cold [60]. For people with COPD, maintaining the WHO warmth guideline of 21°C in living areas for at least nine hours per day was associated with better health status [20]. Donaldson and colleagues also showed a small increase in lung function for patients with COPD, when there was a similar rise in indoor bedroom temperature [26].

Following these associations, Osman and colleagues conducted a small community trial to investigate the effect of increasing indoor temperatures on COPD through home energy improvements. Despite significant implementation barriers, when the improvements were made there was an improvement in respiratory health [21] suggesting a larger trial would be warranted.

## Methods/Design

The Warm Homes for Elder New Zealanders (WHEZ) study is a community-based trial designed to test whether an electricity voucher paid into the electricity account of older people with COPD, with the expressed aim of enabling them to keep their homes warm, results in reduced exacerbations.

Ethical consent was obtained from the New Zealand Multi-Region Ethics Committee MEC/07/05/062 and the Clinical Trial Registration is NCT01627418. Written informed consent was gained from the participants.

### Research questions

The primary research question is whether the provision of an electricity subsidy reduces exacerbations of COPD requiring hospitalisation, or treatment with corticosteroids and/or antibiotics. Secondary questions include whether the dwelling temperature is affected by the electricity intervention and whether the estimated benefits of the intervention exceed the costs.

### Intervention

The intervention is a subsidy of NZ $500 (approximately US $400, £250, €300) towards the household electricity bill, which was calculated as being sufficient for a 2.4 kW electric heater to run continuously for an additional 10 hours a day for 12 weeks, thus raising the temperature of one room substantially, as well as the overall average indoor temperature. The money was paid directly into the household’s electricity account (in the first winter of enrolment for the early group, and the second for the late group). $500 was considered a potentially feasible sum for the government to implement as a national policy and is comparable to the British Winter Fuel Subsidy.

The amount was tied to the electricity bill, as subsidising other common forms of heating, such as wood or liquefied petroleum gas, would potentially have a deleterious effect on indoor air quality, which might adversely affect the participants’ lung function. Initial feedback on this method of subsidy has been positive; there have been very few calls to our phone helpline from people expecting the payment to be made another way.

Due to the complexity of New Zealand’s deregulated domestic electricity industry, which includes competing electricity retailers, organising and implementing the intervention was not a trivial task. However, close liaison with the electricity companies proved worthwhile and most were very co-operative. For example, company representatives, who understood the study, referred back to the study team a request from a participant to withdraw some of the credited money from their account.

### Sample size

The primary outcome of concern was the rate of moderate or severe COPD exacerbations amongst the participants. The TORCH study of combined long-acting beta agonists and corticosteroids found yearly moderate or severe exacerbation rates dropped from 1.13 in the placebo group to 0.85 in the combination therapy group, giving a rate ratio of 0.75 [61]. The study was sized to duplicate a reduction of this size – using a negative binomial model for exacerbation rates as recent analyses have used these, rather than Poisson models, as the negative binomial models better account for individual patient variability [61,62]. A “k” parameter (the shape parameter of the associated gamma distribution) of 0.46 was used as a reanalysis of the TRISTAN data found this the most appropriate (95%CI 0.34-0.60) [62]. These parameters, with a confidence level of 95% and a power of 80% yield an estimated required group size per arm of 239, which allowing for a 15% drop out rate (greater than the 10% found in the *Housing, Insulation and Health study* for the over 65s due to the greater illness of this study population) suggested an initial sample size of 550 participants. If the “k” parameter was 0.4 then the required group size per arm would have been 230, if 0.6 then it would have been 262. If the baseline exacerbation rate was 1.0 rather than 1.13 then the required group size per arm would be 260. Therefore 550 participants was the target sample size.

### Recruitment

From 2009 until December 2010, application forms were distributed through various agencies, including primary health organisations, COPD support groups, public hospital outpatient departments, and community health workers. Letters of invitation were also sent to eligible people discharged after a hospitalisation for COPD. Concerted attempts were made to follow up incomplete application forms, so that literacy difficulties did not automatically disqualify applicants. On receipt of an eligible application form, potential participants were sent an information sheet, personal consent forms for the person with COPD and another adult in the household (optional) and a reply-paid envelope. If the property was rented, consent forms were included for the landlord to agree to retrofitter assessment and insulation.

When the signed consent form was returned, the applicant was sent a letter confirming their acceptance into the study. Where necessary and feasible, ceiling and under-floor insulation were upgraded or retrofitted in homes.

### Wave design and randomisation

As winter was the crucial period for the study, and recruitment is often slow at the start of the process the study was deliberately designed to recruit over two years in ‘waves’. Participants were recruited from three communities from 2009 to early 2011. Those recruited (i.e. consent and baseline data received) by June (early winter in the southern hemisphere) 2010 were assigned to Wave 1, those recruited later were assigned to Wave 2. Each wave was randomised into early and late intervention groups – the early group receiving the intervention the first winter in which the wave was enrolled in the study, and the late group the following winter. Thus *Wave 1- Early* participants received the fuel subsidy during 2010; *Wave 1- Late* and *Wave 2- Early* participants received the subsidy during 2011; *Wave 2- Late* participants received the subsidy during the winter of 2012. The randomisation was carried out by a statistician external to the study team.

The configuration of an autumn baseline with a spring follow-up was chosen in recognition of the significant illness of the sickest participants; with a full winter baseline there could have been more of a bias towards only the least unwell people completing the study. We allowed for a greater drop-out rate than we usually experience due to the illness among the study population.

As the intervention was carried out at a dwelling level, in order to avoid clustering, we selected only one primary participant per household, the person best able to complete the study questionnaire. The baseline interviews were carried out before randomisation so that knowledge of their group allocation could not affect the participants’ measures. In order to utilise the voucher for additional heating, the participants were then informed of their group allocation. Although the interviewers also knew the participant’s group allocation, they were trained to avoid mentioning group allocation status during interactions with the participants to minimise any recall bias. The analyses will be carried out with the researchers blinded to the meaning of the intervention group code. We also included several indicators designed to measure each participant’s propensity to report positive change and will control for the placebo effect by conducting sensitivity analyses using these propensity to positivity scores.

**Figure 1:**
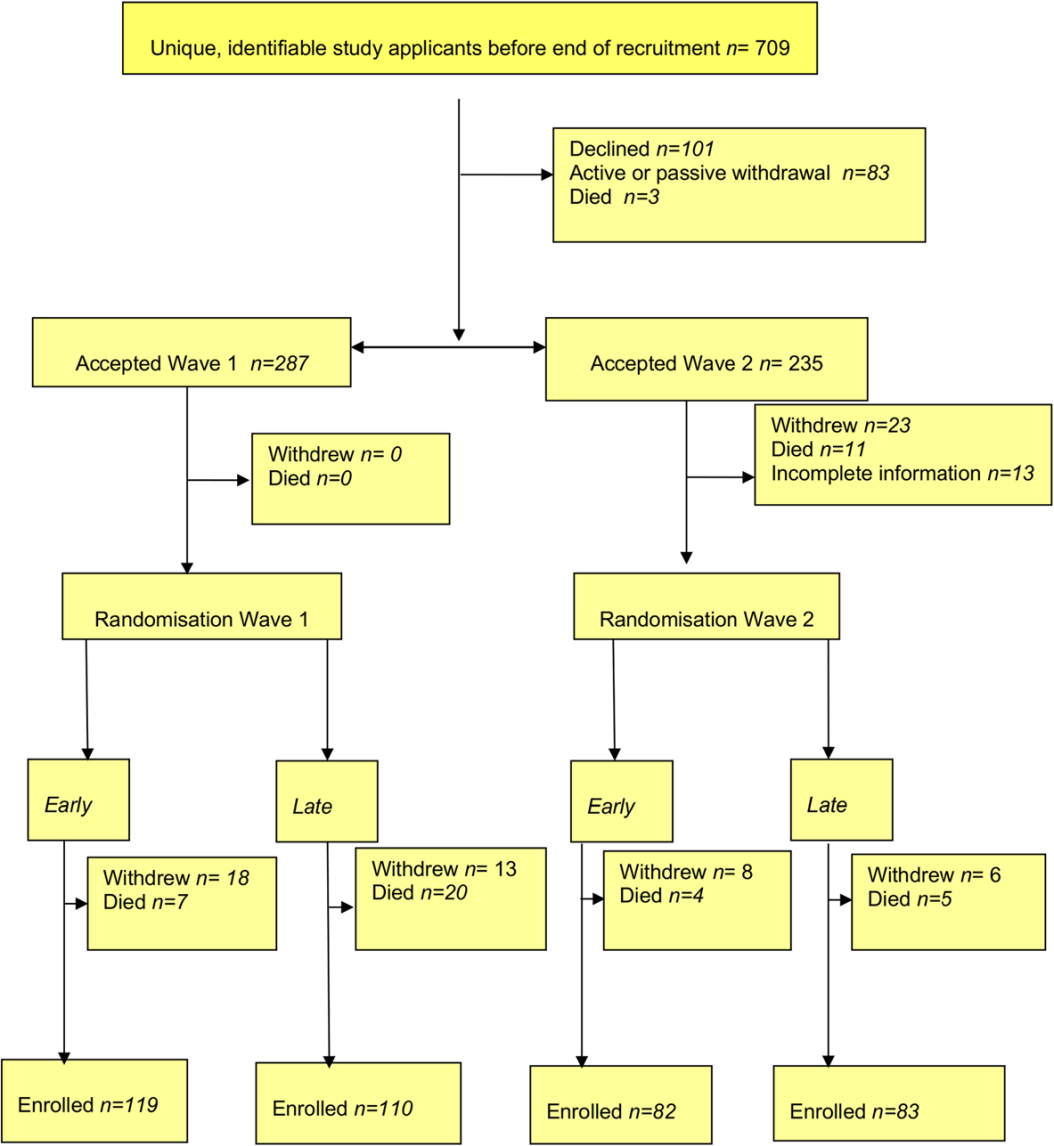
Flow of participants through the study.

**Figure 2:**
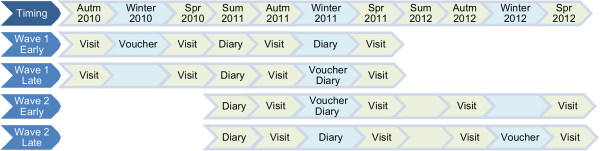
**Study timeline.** The wave design and randomisation meant that the interventions were spread over three years.

The flow of participants through the study is summarised in Figure 1, and the wave design portrayed in Figure 2.

### Participants

The demographic characteristics of the 522 COPD participants enrolled are presented in Table 1; 20% were Māori, 77% New Zealand European or European and 2% Pacific peoples, and 3% reported belonging to other ethnic groupings, with only 3% of people reporting more than one ethnicity. We used the ethnicity classifications recommended by Statistics New Zealand. We succeeded in recruiting an older population (median age 71), with nearly a fifth (18%) of participants being over 80 at baseline.

**Table 1:** Baseline characteristics of participants

**Characteristic**	**% (number)**
General health fair or poor	47% (216/460)
Phlegm most days a week	52% (239/458)
Shortness of breath most days a week	62% (288/461)
4 or more bouts of severe chest trouble over winter	22% (99/457)
Able to walk only 100m or less on the flat before stopping for breath	42% (192/457)
At least one cold/flu over winter	65% (294/453)
Ever told have heart disease	47% (210/446)
Ever told have at least 2 of heart disease, cancer, liver disease, renal disease, diabetes, arthritis	42% (179/430)
On home oxygen	8% (39/460)
Received flu vaccine	84% (387/458)
Received pneumonia (pneumococcal) vaccine	22% (96/420)
Lived in dwelling for more than 9 years	63% (290/463)
Dwelling built before 1977	72% (303/422)
Own the dwelling	76% (350/462)
Dwelling always cold	42% (196/462)
Cold at least partly to keep cost of heating down	48% (213/445)
Shivered inside at least once previous winter	56% (249/445)
‘Dragon breath’ inside at least once previous winter	39% (177/450)
Plug in medical equipment	21% (94/454)
Gave “no other choice” as a reason for main form of heating	43% (193/450)
Gave cheap or easy to budget with as reason for main form of heating	29% (129/450)
Eligible for a high use health card*	60% (251/416)
Eligible for community services card **	77% (337/438)
Receive a disability allowance	51% (220/435)
Currently smoke daily	17% (81/463)
European ethnicity	77% (349/452)
Māori ethnicity	20% (92/452)
Pacific ethnicity	2% (10/452)
Male	50% (228/456)
Aged under 65	27% (122/455)
Aged over 80	18% (81/455)
Median age	71 Years

*This card entitles some frequent users of health services to reduced health co-payments.

** This card entitles people on low income to reduced health (and some other) co-payments.

Initially the intention was to accept people into the study whose post-bronchodilator spirometry results demonstrated GOLD stage II (moderate) COPD or worse (GOLD, 2005). However, the logistics of conducting high quality spirometry in the homes of COPD patients created a recruitment bottleneck, stalling the study progress. Instead, two extra questions^a^ were added to the application form to identify people who had suffered a ‘moderate’ or ‘severe’ exacerbation of COPD as defined by the ATS/ERS Taskforce [63], and were therefore considered at risk of subsequent exacerbations. Thereafter, those who had been admitted to hospital for COPD and/or taken steroids or antibiotics for COPD within the last three years were considered eligible.

Other essential requirements were for applicants to be living in the community, in a household responsible for its own electricity bill, and with the intention of remaining in the same house for the duration of the study. In recognition of the earlier onset of COPD morbidity and mortality for Māori, study participants had to be 55 years of age or older, although applicants of 45 years or over were considered if referred on the specific recommendation of community health workers.

Co-morbidities were not exclusion criteria, unless the co-morbidities rendered the participant unable to communicate effectively. Co-morbidities are to be expected in this age group and, to be effective, an intervention must improve COPD in the presence of co-morbidities. We used a Charlson style index [64-66], modified to be based on self-report rather than ICD codes, to measure co-morbidities. Forty-two percent of baseline respondents reported having been told that they had two or more of: heart disease, cancer, liver disease, renal disease, diabetes and arthritis; 47% reported heart disease.

Using fuels other than electricity for heating was not an exclusion criterion, but participants were informed that only electricity bills would be credited. As we were attempting to model a possible population–level intervention, we wanted to have as broad inclusion criteria as feasible.

All participants were encouraged to be vaccinated annually against influenza, but this vaccination had to be obtained through their own GP or respiratory physician as part of the standard health-promotion campaigns. Eighty-four percent of baseline participants reported having had a flu injection for the most recent flu season and 22% reported a pneumococcal vaccination in the last five years.

In 171 households a support person also provided baseline information. These people completed a short questionnaire on the burden (if any) of providing care for the COPD participant, as well as the carer’s general health.

### Setting

The study was set up in three predominantly low-income urban areas. This study was carried out in partnership with local organisations with an interest in health and housing [44]. These organisations included a Māori health provider, a District Health Board, and Primary Health Organisations. Two communities were in the lower North Island (Whanganui and Wellington) and one in the South Island (Christchurch). Contracts were negotiated with these providers, who then worked closely with the research team, employing local field workers, nurses or community nurses, trained to conduct spirometry and administer the questionnaires.

One of the community organisations, Tu Kotahi, a Māori Asthma and Respiratory provider operating under the umbrella of Kokiri Marae, a large urban-based *marae* (communal meeting place), has participated in our two previous community housing trials. Our partnership is seen as resulting in many positive outcomes for both parties. For the Tu Kotahi Māori community it has brought insulated homes, improved heating, minimising the effects of allergens and increased information about healthy housing; for the University researchers it has provided access to the considerable knowledge of the community, as well as capacity building for both individual researchers and research groups.

Tu Kotahi’s involvement in housing research projects has highlighted the effects of poor housing on the health of their *whānau* (extended family community). In 2007, Tu Kotahi established a Māori COPD support programme and an important aspect has been the focus on healthy housing. Being research partners in this study has enabled Tu Kotahi to work more closely with *whānau* in their homes to assess their health status prior to, during and following the winter period, which has brought benefits beyond the study participation.

“The opportunity to follow up utilising our own community health worker has been crucial to maintaining the majority of *whānau* on the study. We have experienced a transient population during the study and this has often meant that we have only been able to locate *whānau* with information provided by the community health worker’s networks. Often, we discover that [members of the] *whānau* have been hospitalised or they have had to travel home for *tangihanga* (funerals). We also discovered how socially isolated a number of *whānau* are, this sometimes involved numerous visits before the door was opened for the community health worker to complete the study questionnaire and tests. We were also saddened at the number of *whānau* who passed away during the study; many of the *whānau* who participated experienced other co-morbidities that impacted on their respiratory conditions.”

The South Island provider, Community and Public Health, is the public health unit for the region of Canterbury. Staff also had a well-established working relationship with the researchers. Since 2001, these staff also carried out their own local health promotion strategies connected to housing improvement. Working with the researchers was therefore considered a mutually worthwhile undertaking in terms of developing opportunities for public health action.

In September 2010 the first of a continuing series of earthquakes and aftershocks occurred in Christchurch and the surrounding area [67]. With considerable fortitude the Christchurch team did not let the earthquake, aftershocks or widespread liquefaction disrupt the spring visits by the field workers. In February 2011 a major earthquake caused a massive collapse of buildings and 185 lives were lost [68].

Workplaces in the CBD were severely damaged and the area was closed to the public. The city’s infrastructure was in chaos as hundreds of organisations and businesses had no premises to work from, including the staff of Community and Public Health. This had an impact on the WHEZ study in a number of ways and the community researchers set about trying to overcome the subsequent problems.

Researchers agreed it was necessary to postpone interviewing for a three-week break to re-establish basic infrastructure and solve serious logistical problems. Laptops and other research equipment were initially out-of-bounds in the damaged office and only salvaged much later; however, the main computer system remained intact and data could be accessed remotely. The researcher’s home served as the research hub for the next year. There was only minimal disruption to the household visits when a further round of massive aftershocks and liquefaction occurred in June 2011. While the seismic activity has continued, it has diminished in intensity.

There was damage to the homes of the field workers and many participants, including chimney damage making fireplaces unusable, and structural damage affecting homes’ weather-tightness. There was also significant damage to infrastructure such as water, power lines and sewerage. For instance, because of damaged sewer lines in her neighbourhood, one field worker had to rely on a portable toilet on her street front for many months.

Some participants in the WHEZ study vacated their houses to a safer place for a period of time. Some moved to the less affected western suburbs or moved out of Christchurch for a number of weeks until they felt it was safe to return and this made it difficult for field workers to track participants down. Driving to the participants’ homes became laborious due to the extensive damage to roads. The study protocol stating that anyone who moved could not remain in the study was modified for the Christchurch participants; because so many other housing related variables had changed for many of the participants, there was little point in insisting on address stability. We also did not think it ethically appropriate to withdraw the fuel subsidy from participants who were forced to move because of the earthquake, but were otherwise keen to continue with the study.

The earthquake and continuous aftershocks were an emotionally traumatic experience for the participants, field workers and the co-ordinator. Financial worries about damaged housing, fear, and uncertainty about personal safety all contributed to this. The arrival of winter heightened people’s fears about the adequacy of the damaged electricity network to supply enough energy for households to be warm in winter. The field workers commented on the fears expressed to them during the research visits. The authorities were contacted for help in some desperate cases of damaged homes and heaters. Over the region, 9,000 new heaters were installed in the homes of people who had lost their primary heat source, and were paid for by the householder’s insurance company.

### Data collection

#### Spring and autumn questionnaires

The first main data collection commenced in autumn (2010 for Wave 1, 2011 for Wave 2). The participants’ basic anthropomorphic measures were collected, spirometry conducted and a health questionnaire completed. Height was measured using portable stadiometers (Seca 214), and weight with Tanita TIHD316 scales.

Pre- and post- bronchodilator spirometry was carried out, following ATS/ERS 2005 Task Force Guidelines [69], using a portable ultrasonic spirometer (Easy One™). Subjects performed the tests seated. All community workers were trained to perform spirometry to ATS/ERS standards. FVC, FEV_6_, FEV_1_, PEFR, and FEF_25-75_ were recorded. Participants were encouraged to perform three acceptable manoeuvres, within a maximum of eight attempts. Participants who refused to attempt spirometry were not excluded from the study. Post-bronchodilator spirometry was conducted 30 minutes after the administration of 400 micrograms salbutamol (Ventolin®) via a Volumatic® spacer.

The questionnaire for the primary participant included sections on respiratory health, health care usage, general health, co-morbidities and quality of life, as well as tenure, heating used in the dwelling and house quality.

Similar data, except for height and weight, were collected during subsequent spring and autumn data collections. One or more data-loggers (HOBO or iButton®) monitoring the temperature (and, for a subset of dwellings, humidity) in the dwelling were placed in winter and collected in spring.

If another adult was living in the dwelling they were also invited to fill in a shorter ‘support person’ questionnaire, including sections on respiratory health, general health and the effects on caregivers of caring for the person with COPD on their life.

#### Summer and winter diaries

Participants were asked to fill in over two-week periods during summer and winter, a detailed diary asking about activity, symptoms, medication, and medical care used.

#### Other data

Objective data on health care usage were found using participants’ National Health Index (NHI) to access hospitalisations. Primary care data were obtained from Primary Healthcare Organisations directly and via the Healthlink network. Objective assessments of electricity usage were obtained from electricity companies (Table 2).

**Table 2:** Outcome measures

	
Primary outcome measure	Number of Moderate and severe exacerbations of COPD
Secondary outcome measures A	Severe exacerbations of COPD for which hospitalisation (ICD-10 code: J44) is required to treat the exacerbation;
	Moderate exacerbations of COPD; defined as requiring treatment with systemic corticosteroids and/or antibiotics.
	Hospitalisations for key lower respiratory tract illnesses (ICD-10 codes: J40-J47)
	All-cause hospitalisation
	Temperature in the living and bedrooms
	Electricity usage
	Costs to health care system
Secondary outcome measures-B	Self-reported quality of life
	Respiratory health of other people living in household
	Any changes in COPD severity (i.e. change in FEV1)
	Study withdrawals due to death or greater dependency
	Net benefits (benefits relative to costs) of the intervention.
	Support person burden

#### Outcome measures

**Acceptability of methods** The overall feedback from the majority of the participants in the WHEZ Study has, to date, been very positive. One such indicative report from a Wellington interviewer described her assessment of participants’ experiences in more detail:

“Although at the initial visit some participants had concerns and were unsure about the study, once they were given more information and further explanation of the study then they willingly and happily participated. In the first year pre-winter visits most of the participants reported how cold and damp their homes were and with the escalating cost of electricity they had to minimize how often they could have the heaters on to warm their homes. In the post–winter visit, the first wave of participants that received the subsidy of $500 commented how grateful and thankful they were for the subsidy because it helped tremendously - especially with the high winter electricity power bills. They also reported on the change in warmth in their homes since they had been insulated.”

### Data analysis

Generalised linear models will be fitted to the data. Covariates potentially included in the model will be study arm, current medication, age, sex, region, baseline severity, smoking status and immunisation status (influenza and pneumococcal). The appropriateness of a Poisson or negative binomial model of exacerbations will be confirmed by examining the residuals of the observed data fitted to the postulated models against an envelope of residuals from a set of simulated trials. If the observed residuals fall inside the simulated envelope then the model is considered to fit adequately. A non-parametric test – rank analysis of covariance – will be used to confirm differences between the groups.

#### Cost-benefit analysis

The design of a cost-benefit analysis of this study is straightforward and follows similar studies’ methods [70,71]. The cost side of the intervention follows from providing a $500 voucher for electricity (and knowing the amount of electricity actually used). On the benefit side, the principal quantifiable outcome is reduced hospitalisations (ideally, exacerbation-reduced or exacerbation-free years would be measurable, but this was beyond the scope of the study). In addition, the study will estimate and value changes in pharmaceutical usage.

## Discussion

COPD is a growing problem in New Zealand and internationally, particularly as there are few proven effective interventions to reduce exacerbations. Although cold housing has been linked to respiratory illness, and respiratory infections linked to COPD exacerbations, only one previous study has directly examined the effect of indoor temperatures on COPD patients.

Being in a cold house during winter is likely to be a risk factor for COPD exacerbations. Many New Zealand households are living in fuel poverty with winter indoor temperatures regarded as unacceptable in other countries. In the WHEZ study we are addressing the houses’ thermal qualities and the effective cost of fuel. We have retrofitted insulation into the houses of vulnerable older people, which we have previously shown is an effective intervention to reduce fuel bills and increase the indoor temperature and in this study we are also subsidising the price of electricity, which indirectly has an impact on disposable household income.

While our intervention is modelled on the age-eligibility of the British Winter Fuel Allowance (households in which there is at least one person over 60), it is targeted and only includes older people who *also* have a chronic respiratory illness. In Britain a cash payment is made, whereas our intervention is paid directly into the participant’s electricity account and being in the southern hemisphere, these payments are *not* made just before Christmas and the holiday season. These factors make it more likely that the participants will spend the payment on additional heat.

New Zealand has no income support directly analogous to the Winter Fuel Allowance; however, it does have a heating supplement available to some people who receive a disability allowance, but this is only available to people on low income, who have regular on-going costs, above those of average people because of their disability or illness. Due to strict eligibility requirements, it is often difficult for low-income people to access this benefit and buy extra warmth as indicated by almost half the vulnerable participants in this study stating that they were colder than they would have liked because of the cost of heating; just over half had shivered inside the previous winter.

The WHEZ study is a staggered intervention community trial, which does not ‘blind’ the participants as to whether they are in the intervention or the control group. When participants were informed that they would be receiving the fuel subsidy, they were encouraged to see “heat as your medicine” and provided with sample calculations to help them estimate how much heating they could use without overspending the payment. The study is designed to explore the extent to which this supplement is effective for helping older people to maintain adequate indoor environments.

## Conclusions

We are successfully implementing a multi-centre, community-based, randomised control trial in partnership with community agencies and primary health organisations to see whether an electricity voucher enables elderly people with COPD to better heat their homes, and in turn, whether this reduces exacerbations of COPD symptoms, and hospitalisation.

The implementation has been complex and partly disrupted by a massive seismic event affecting the Christchurch region. However, the WHEZ study shows that it is possible to evaluate the merits of targeted welfare benefits for the health of people with chronic and debilitating diseases such as COPD, for whom an inability to keep warm is a common problem.

## Endnotes

^a^ When was the last time your COPD got so bad you needed to go to hospital? and b)When was the last time your COPD got so bad you needed to take antibiotics or steroids? The options for both questions were: Never/In the last year/1-3 years ago/ More than 3 years ago.

## Abbreviations

COPD: Chronic Obstructive Pulmonary Disease; WHEZ: Warm Homes for Elder New Zealanders (study)

## Competing interests

The authors declare that they have no competing interests.

## Authors’ contributions

PHC overall oversight; PHC, HV, TI, RC, CD, NP, MB & JC wrote application and study flow; HV, HW day to day oversight; GP, CD, AC data collection in different areas; TI, JC respiratory oversight; JZ, MB COPD analysis; all contributed to paper and writing. All authors read and approved the final manuscript.

## Pre-publication history

The pre-publication history for this paper can be accessed here:

http://www.biomedcentral.com/1471-2458/13/176/prepub
